# Blue Light Effect on Metabolic Changes in Induced Precocious Puberty in Rats

**DOI:** 10.3390/biology14080951

**Published:** 2025-07-28

**Authors:** Luciana-Mădălina Gherman, Elena-Mihaela Jianu, Ștefan Horia Roșian, Mădălin Mihai Onofrei, Lavinia Patricia Mocan, Veronica Sanda Chedea, Ioana Corina Bocsan, Dragoş Apostu, Andreea Roxana Todea, Eva Henrietta Dulf, Emilia Laura Mogoșan, Carmen Mihaela Mihu, Cătălina Angela Crişan, Ștefan Cristian Vesa, Anca Dana Buzoianu, Raluca Maria Pop

**Affiliations:** 1 Experimental Centre, “Iuliu Haţieganu” University of Medicine and Pharmacy, Louis Pasteur, No. 6, 400349 Cluj-Napoca, Romania; luciana.gherman@umfcluj.ro; 2 Academy of Romanian Scientists, Ilfov 3, 050044 Bucharest, Romania; emiliamogosan@yahoo.com (E.L.M.); raluca.pop@umfcluj.ro (R.M.P.); 3 Histology, Department of Morphofunctional Sciences, “Iuliu Haţieganu” University of Medicine and Pharmacy, Victor Babeș, No. 8, 400012 Cluj-Napoca, Romania; madalinonofrei99@gmail.com (M.M.O.); lavinia.trica@gmail.com (L.P.M.); carmenmihu2004@yahoo.com (C.M.M.); 4 “Niculae Stăncioiu” Heart Institute Cluj-Napoca, 19-21 Calea Moților Street, 400001 Cluj-Napoca, Romania; dr.rosianu@gmail.com; 5 Department of Cardiology—Heart Institute, “Iuliu Haţieganu” University of Medicine and Pharmacy Cluj-Napoca, Calea Moților Street No. 19-21, 400001 Cluj-Napoca, Romania; 6 Research Station for Viticulture and Enology Blaj (SCDVV Blaj), 515400 Blaj, Romania; chedeaveronica@yahoo.com; 7 Pharmacology, Toxicology and Clinical Pharmacology, Department of Morphofunctional Sciences, “Iuliu Haţieganu” University of Medicine and Pharmacy, Victor Babeș, No. 8, 400012 Cluj-Napoca, Romania; bocsan.corina@umfcluj.ro (I.C.B.); abuzoianu@umfcluj.ro (A.D.B.); 8 Orthopaedics and Traumatology, Department of Surgical Specialities, “Iuliu Hatieganu” University of Medicine and Pharmacy, Victor Babeș, No. 8, 400012 Cluj-Napoca, Romania; apostudragos@yahoo.com; 9 Department of Automation, Faculty of Automation and Computer Science, Technical University of Cluj-Napoca, Memorandumului Street No. 28, 400014 Cluj-Napoca, Romania; andreea.todea@student.utcluj.ro (A.R.T.); eva.dulf@aut.utcluj.ro (E.H.D.); 10 Pathophysiology, Department of Morphofunctional Sciences, Faculty of Medicine, “Iuliu Hațieganu” University of Medicine and Pharmacy Cluj-Napoca, 400012 Cluj-Napoca, Romania; 11 Department of Neurosciences, “Iuliu Haţieganu” University of Medicine and Pharmacy Cluj-Napoca, 400012 Cluj-Napoca, Romania; ccrisan2004@yahoo.com

**Keywords:** blue light, precocious puberty, circadian rhythm, leptin, glucose, insulin, cholesterol, triglyceride

## Abstract

Modern life, characterized by constant exposure to artificial light from electronic devices, such as light-emitting diodes (LEDs), disrupts the natural circadian rhythm and induces important metabolic changes. The impact of blue light exposure on male and female rat’s onset of puberty, hormonal and biochemical parameters was assessed by comparison between the four study groups: the control group (CTRL) maintained under normal light conditions, the group exposed to blue light from a mobile phone (MP), the group subjected to blue light from a computer screen (PC), and the group exposed to blue light from an LED lamp (LED). Both female and male rats exposed to PC and LED failed to thrive, with a significantly lower body weight intake than the CTRL group. All three distinct sources of blue light interfered with the cyclicity of the estrous cycle in female rats. A marked decrease in the number of complete estrous cycles and the highest incidence of incomplete cycles were noticed in the LED group. Elevated ALT, AST, glucose, and insulin levels were influenced in a gender-specific manner, and depending on the source of emitted light. Prolonged blue light exposure induces significant metabolic disruptions and possesses important future research potential in identifying explicit pathways regarding this environmental stressor.

## Simple Summary

The increasing use of digital devices has raised concerns about the effects of blue light on children’s health, especially during critical stages of development. This study explored how exposure to blue light may influence early puberty and metabolism. Using a model of induced early puberty in young female rats, we examined how different durations of blue light exposure affected physical and hormonal changes. The results showed that blue light accelerated the onset of puberty and caused changes in body weight and hormone levels. These findings suggest that extended exposure to blue light—similar to what children experience from screens—may contribute to early puberty and affect long-term health. This research highlights the need for greater awareness about screen time and its potential impact on growing children. Understanding these effects can help families and healthcare professionals make informed choices to protect healthy development.

## 1. Introduction

Modern life exposes organisms to continuous artificial light emitted by electronic devices such as smartphones, tablets, computer screens, and LED lamps. Among these, blue light—characterized by short wavelengths (~480 nm)—has been increasingly recognized for its biological impact beyond visual processing. Previous studies have highlighted that prolonged exposure to blue light can lead to significant metabolic alterations, particularly in animal models [1,2]. These effects include disruptions in glucose homeostasis, lipid metabolism, hormonal regulation, and energy balance, which may occur independently of circadian rhythm modulation [3,4,5]. Experimental studies on rodents have shown that blue light exposure during the active or rest phase results in increased body weight, elevated blood glucose and insulin levels, and altered lipid profiles, including reduced HDL and increased triglycerides [6,7]. Furthermore, hepatic enzymes such as ALT and AST tend to be elevated following chronic blue light exposure, suggesting potential liver stress or dysfunction [8]. These metabolic changes are often accompanied by modified leptin secretion, indicating altered appetite control and adipose tissue signaling [9]. The mechanism by which blue light induces such metabolic effects is thought to involve direct and indirect pathways, including oxidative stress, systemic inflammation, and modulation of peripheral clock genes involved in metabolism [8,10]. Notably, sex-specific responses have also been documented, with female and male animals exhibiting different susceptibilities to blue light–induced metabolic disruption, particularly regarding hormonal balance and energy storage [11].

At the global level, the pandemic crisis represented a critical period for each individual. Throughout this period, there have been major changes in everyone’s lifestyle, including prolonged exposure to different devices, a sedentary lifestyle, changes in the circadian cycle, and improper nutrition. All these changes have determined an increase in the number of obesity cases, regardless of age, and early development of puberty is highlighted in the case of young children [11]. The widespread adoption of LED technology, characterized by its long lifespan, energy efficiency, and low cost, has revolutionized the lighting sector. However, the spectral composition of LEDs, enriched with a significant proportion of blue light, gives them a distinct impact on the human circadian rhythm. Compared to other artificial light sources, LEDs emit approximately twice as much blue light, excessively stimulating the retinal photoreceptors sensitive to blue light. This chronic exposure, especially during the evenings, can disrupt the endogenous synchronization of the sleep-wake cycle by inhibiting the secretion of melatonin, a key hormone of the circadian rhythm. The widespread use of blue-light-emitting electronic devices, such as computers, mobile phones, and tablets, amplifies this effect, with significant implications for sleep health and well-being of adolescents [12]. The intensive integration of mobile technologies into the lives of adolescents has generated profound changes in the media consumption patterns and social interactions of this age group. Smartphones, with their multiple functionalities, have become extensions of adolescents’ digital identities, facilitating access to an unlimited amount of information and entertainment. Research indicates that American adolescents spend a significant number of hours consuming video content on these devices, and internet addiction, with a penetration of almost 95% among this cohort, underlines the central role of mobile phones in the daily lives of young people [13].

In modern life, LED technology has become ubiquitous, being used in various electronic devices [14]. This, in addition to benefits such as energy efficiency and long life, can represent a threat to circadian rhythm (CR). Prolonged exposure to blue light that causes CR disruption may contribute to the aetiology of metabolic syndrome through the impact on energy metabolism and glucose regulation [15]. Studies using animal models link prolonged light exposure to disturbances in glucose homeostasis, body weight regulation, and metabolic disorders, all consequences of CR disruption [2,16,17].

Precocious puberty (PP) refers to the premature onset of secondary sexual characteristics, defined as breast development in girls under 8 years of age or growth of testicles and/or pubic hair in boys under 9 years of age. Precocious puberty can be classified into two main categories: central or true precocious puberty (CPP), caused by premature maturation of the hypothalamic–pituitary–gonadal axis, and peripheral precocious puberty (PPP), which results from excessive production of sex hormones, independent of gonadotropin secretion (also known as precocious pseudopuberty) [18]. Puberty, a period of hormonal, behavioural, and physical changes leading to reproductive competence, occurs in both humans and rodents like rats [19]. The onset of puberty in both models has shifted due to genetic predisposition, environmental factors, stress, and pollution. Notably, light exposure, particularly blue light (450–470 nm wavelength), contributes to this variability [20,21]. Previous literature studies demonstrate that adult female animals exposed to continuous light exhibit persistent or irregular estrous cycles, sometimes leading to sterility [22,23,24]. The vaginal opening, a critical anatomical landmark in female rat reproductive development, signifies the beginning of the estrous cycle. Vaginal smears, analyzed for predominant cell types, are the most common method for estrous cycle stage determination [25,26]. In males, preputial separation, marked by the separation of the prepuce from the gland penis, marks the onset of puberty [27,28]. Consequently, estrous cycle irregularities and timing of the first complete cycle are used as indirect markers of hypothalamic–pituitary–gonadal axis activation, relevant in pseudopuberty models. To date, previous studies have shown that chronic blue light exposure accelerates puberty onset and induces metabolic disturbances in female rats [29,30] and in male rats, and causes precocious puberty in male rats, with suppression of spermatogenesis, vasodilation in testicular interstitial areas, and disruption of basement membrane integrity [31]. Furthermore, convolutional neural networks were used to classify estrous cycle stages based on vaginal smear images [30].

Thus, the primary objective of this study was to investigate whether prolonged blue light exposure from widely used electronic devices induces metabolic changes, specifically on lipid and glucose metabolic markers, in a murine model (rats) of induced precocious pseudopuberty secondary to blue light exposure. The secondary aim was to investigate the cumulative physiological effects of blue light exposure from the different digital devices (phones, computer monitors, and LED lamps) to assess whether the source of blue light and the biological sex of the animals modulated the observed outcomes.

We hypothesize that prolonged blue light exposure induces early pseudopuberty onset in this model. We examined whether this exposure triggers stress responses, affecting metabolism in both sexes.

Artificial intelligence (AI) solutions have been widely used in healthcare in the last few years. Recent developments in deep neural networks have contributed to significant advances in medical image processing. Much ongoing research is aimed at helping medical practitioners by providing computer-aided systems to analyze images and diagnose diseases. Our study aimed to build on this evidence by modeling the cumulative exposure to blue light from multiple devices to simulate modern sedentary and screen-dominant lifestyles. Additionally, we aimed to evaluate sex-specific susceptibility to blue light exposure and to identify cytological features uniquely associated with blue-light-induced precocious pseudopuberty, extending beyond the conventional assessment of estrous cycle staging.

## 2. Materials and Methods

### 2.1. Animals

The animals used in this study were provided by the authorized sanitary and veterinary unit of “Iuliu Hațieganu” University of Medicine and Pharmacy Cluj-Napoca, Romania. The animals were held under a controlled microclimate (21–24 °C and 40–45% humidity) benefiting from food and water “ad libitum” for the first 21 days of the animals’ life, following the optimal conditions of the care in line with the Principles of Laboratory Animal Care (NIH publication No. 86-23, revised 1985) and adhered to the ARRIVE guidelines.

A total of 64 Wistar rats (32 males and 32 females), aged 21 days postnatal (PND 21), were included in the study. Animals were stratified by sex (n = 8 per group) and housed in groups of four per cage. The rats, initially weighing between 30 and 50 g, were maintained under a 16-hour light/8-hour dark cycle for the 30-day duration of the experiment, to mimic prolonged light exposure typical for screen usage [32]. The “Iuliu Hațieganu” Ethics Committee of the University of Medicine and Pharmacy and the Sanitary-Veterinary and Food Safety Directorate from Cluj-Napoca authorized all conducted procedures, which were carried out under veterinary sanitary rules (permission number 380/25.08.2023).

### 2.2. Experimental Design—LED Exposure Conditions

**Figure 1:**
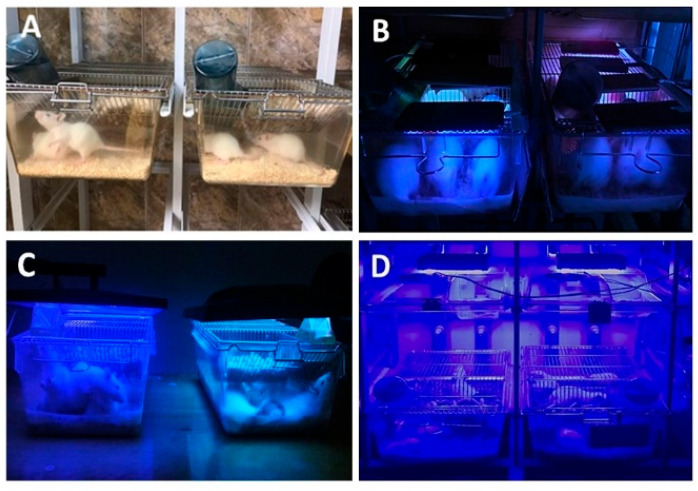
(**A**)—Control group (CTRL) exposed to the artificial light, (**B**)—group exposed to the blue light from mobile phone (MP), (**C**)—group exposed to the blue light from computer screen (PC), and (**D**)—group exposed to the blue light from the led lamp (LED).

To mimic the children/adolescents’ exposure to blue light conditions encountered in their daily lives from different devices (e.g., mobile phones and computer screens), we used the following experimental protocol. Rats were randomly assigned to four male groups and four female groups. These groups included a control group (CTRL) exposed to artificial light conditions (warm white laboratory fluorescent lighting 3000 K) (Figure 1A), a group exposed to blue light from a mobile phone (MP) (Figure 1B), a group exposed to the blue light from a computer screen (PC) (Figure 1C), and a group exposed to the blue light from a LED lamp (LED) (Figure 1D). The experimental protocol included positioning two light sources (4 phones/cage, one monitor/cage), at 15 centimetres above the animal subjects, respectively, one LED lamp/cage at 1 m above the animal subjects as a standard procedure to avoid overheating and ensure uniform exposure.

The control group was maintained under standard warm white fluorescent lighting (~3000 K), which emits light with a correlated color temperature (CCT) resembling natural incandescent light. This type of light has minimal blue spectrum emission and is commonly used in laboratory settings to avoid disrupting rodents’ circadian rhythms. The light intensity at cage level was approximately 150 lux, which falls within the range recommended for standard housing laboratory rats under non-disruptive conditions.

In this study, animals were exposed to three distinct artificial light sources emitting blue light: a smartphone (iPhone 11), a computer monitor (Dell SE2422H), and an LED lamp. The iPhone 11 was configured to operate at maximum screen brightness, with a luminance of approximately 625 cd/m^2^, resulting in an estimated illuminance of ~400 lux at a 15 cm distance. Although not equipped with high-brightness HDR capabilities found in newer Pro models (which can exceed 800–1000 cd/m^2^), this device emits a significant spectral peak in the blue wavelength range (~455 nm), characteristic of W-LED backlighting. The Dell SE2422H monitor was similarly operated at full brightness. It has a typical peak luminance of 250 cd/m^2^, corresponding to an estimated illuminance of ~160 lux at 15 cm. The monitor features a VA panel with W-LED backlighting and covers approximately the full sRGB color space, offering a typical spectral profile for this type of display. An LED lamp was specially designed for this experiment, operating at its maximum intensity throughout the study period. The illuminance was measured at 395 lx, while the lamp’s peak luminance was 398 cd/m^2^. The emitted light predominantly corresponds to a wavelength of 450 nm, placing it in the blue region of the visible spectrum. To maintain consistent exposure conditions, all automatic brightness adjustment settings (e.g., ambient light sensors, screen dimming, or sleep modes) were disabled on both devices. Illumination was continuous throughout the exposure period, ensuring stable emission parameters during the experimental sessions.

To evaluate the impact of light exposure, all experimental groups were subjected to a standardized lighting regimen, consisting of light exposure from 07:00 a.m. to 11:00 p.m., followed by a dark period from 11:00 p.m. to 07:00 a.m. Experimental groups were exposed to blue light sources only during the 16 h light phase. All groups had the same photoperiod, differing only in light source. All three blue light sources emitted radiation within a narrow wavelength range of 415–470 nm.

### 2.3. Body Weight Measurements

The experimental design included an initial assessment of the body weight of all animals on day 0, corresponding to PND 21. To monitor body weight dynamics over the course of the experiment, measurements were conducted every 3 days in both female and male rats during the 30-day exposure period.

### 2.4. Sample Collection

To investigate the clinical implications of this study, a longitudinal experimental design was employed. Blood samples were collected from rats at three distinct developmental stages: postnatal day 21 (PND 21), PND 36, and PND 51. To enhance translational relevance to human models, blood draws were standardized and performed via caudal vein puncture between 9:00 and 10:00 a.m. All plasma samples were meticulously collected and stored under optimal conditions to preserve biomarker integrity.

Concurrently, the estrous cycle of female rats was monitored through daily vaginal smear analysis. To ensure accurate estrous cycle staging, daily vaginal smears were collected from female rats, beginning with the vaginal opening and continuing until postnatal day 51 (PND 51). All collections were standardized to the 9:00–10:00 a.m. timeframe to minimize potential variability in estrous stage identification.

### 2.5. Puberty Assessment

**Figure 2:**
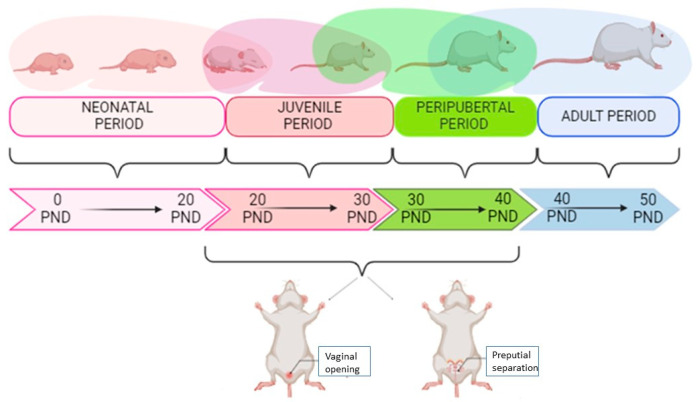
Developmental stages in female rats and indicators of pubertal onset.

The chronological progression of female rat development, divided into four main periods: neonatal (PND 0–20), juvenile (PND 20–30), peripubertal (PND 30–40), and adult (PND 40+) (Figure 2). Pubertal onset occurs during the peripubertal period, typically between postnatal day (PND) 30 and 40, and is marked by two key physiological events: vaginal opening and preputial separation. In females, vaginal opening (VO) is a widely accepted external marker of pubertal onset and occurs spontaneously as a result of rising estrogen levels. VO was monitored daily, and its occurrence was recorded as a primary endpoint in the assessment of puberty timing. This indicator is both non-invasive and reliable for evaluating the effects of treatments or environmental factors on sexual maturation in rodent models. The diagram highlights the timeline and visual cues associated with these changes, supporting the detailed characterization of developmental stages in this study.

Sexual maturation was assessed by recording vaginal opening and monitoring estrous cyclicity in females, as well as evaluating preputial separation in males [33] (Figure 2). Vaginal opening in Wistar rats typically occurs between PND 30 and 40, while preputial separation occurs around PND 44–48.

### 2.6. Estrous Cycle Assessment

**Figure 3:**
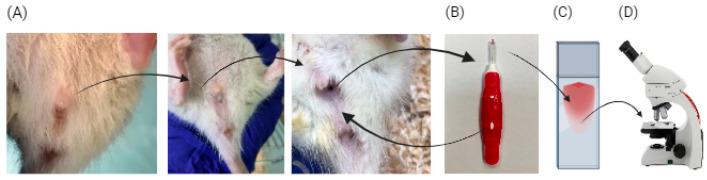
Assessment of vaginal opening (**A**), collection of vaginal smears (**B**), smear fixation (**C**), and estrous cycle stage determination (**D**).

Estrous cycle evaluation was performed through daily vaginal cytology for 30 days. Vaginal smears were collected within a consistent time window (9:00–10:00 a.m.) to minimize the risk of missing a stage due to the brevity of estrous cycle phases. The estrous cycle in rats lasts approximately 4–5 days on average and consists of four distinct phases: proestrus, estrus, metestrus, and diestrus. Each phase is characterized by specific hormonal and cytological changes that can be monitored through vaginal smear analysis [34,35]. A complete cycle is defined by the presence of at least three consecutive stages, including a return to proestrus, which indicates normal ovarian function. In contrast, fragmented or irregular stages suggest an incomplete or disrupted cycle [25]. A sterile interdental brush, with a size of 0.4 mm diameter, was inserted into the vagina and rotated clockwise to gently collect exfoliated cells. The collected material was then spread in a very thin layer on the surface of a clean histological slide and air-dried for 3–5 min (Figure 3). Subsequently, the smears were stained using the Pap stain protocol, a standard technique employed in human cytology [25]. The process of Pap stain staining involved various staining solutions and reagents to create a multichromatic effect. Specimens were initially fixed in an alcohol-based preservative solution to preserve the morphology of the cells. After undergoing hydration steps, the Pap stain was sequentially applied, typically using three solutions: hematoxylin for nuclei, an orange G solution for cytoplasm and background staining, and an eosin-based solution for cytoplasmic component differentiation. Each stain required different incubation times and rinses, as well as differentiators to regulate the color intensity and to achieve the optimal contrast. Finally, the stained smears were dehydrated, cleared, and mounted for microscopic examination (Figure 3).

The slide evaluation was first performed at a low-power (10× objective) scan to provide an initial overview. Subsequently, higher magnifications (20× and 40× objectives) were employed to evaluate cellular details. To ensure inter-observer consistency in case of disagreement, slides were evaluated simultaneously by multiple observers. The estrous cycle stage was then assigned based on the predominant cellular morphology observed in over 50% of the cells.

### 2.7. Assessment of Estrous Cycle

**Figure 4:**
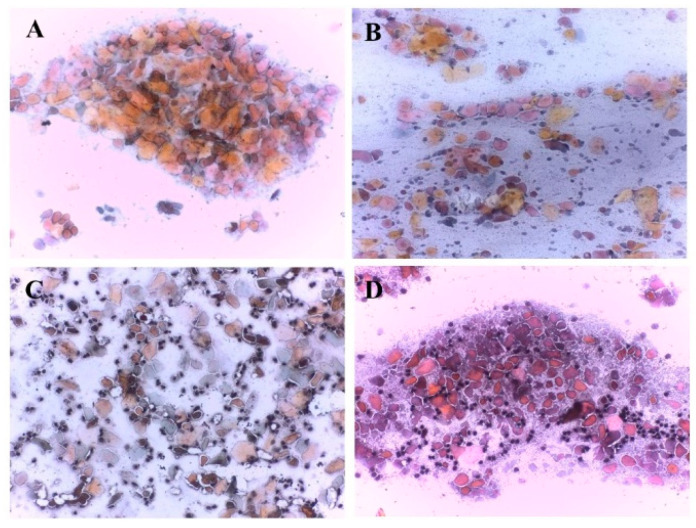
The histopathological aspect of the four stages of the estral cycle: proestrus (**A**), estrus (**B**), metestrus (**C**), and diestrus (**D**).

This study examined vaginal cytology samples to pinpoint the estrous cycle stage (Figure 4). The presence or absence, size, and ratio of various cell types, particularly squamous epithelial cells and neutrophils, were taken into consideration. While the squamous cells displayed varying degrees of cornification, reflected by their orange, pink, or blue colouring, cell morphology played a more critical role in stage determination. Specifically, the presence of small, round neutrophils indicated the later stages (metestrus or diestrus). All samples collected within the initial 10 days met the evaluation criteria due to an adequate number of squamous cells. However, a decline in squamous cells was observed from day 10 onwards, reaching depletion by the final collection (20 days). The first stage of the estrous cycle is represented by proestrous (Figure 4A). In this stage, the distinctive sign is represented by nucleated squamous cells, small, round, and homogeneous, grouped and densely packed, showing round nuclei, large compared to the cell cytoplasm. Rare neutrophils and anucleated epithelial cells could also be present. The cells representative of the second stage of the estrous cycle (Figure 4B), respectively, estrus, are anucleate epithelial cells, which present various shapes (folded or wrinkled) and are dispersed diffusely on the surface of the slide. Along with these, rare phantom nuclei as well as keratin sticks were observed. In the metestrus stage (Figure 4C), polymorphonuclear neutrophils infiltrate among the anucleated epithelial cells, forming an ensemble of neutrophils and scales, representative of this stage. The last stage of the estral cycle is represented by diestrus (Figure 4D), which is characterized by the presence of neutrophils, nucleated and anucleated squamous cells, being present in decreasing order of their frequency. The cell size and shape, as well as the cytoplasmic amount, show considerable variations, from round to polygonal and irregular or even angular (Figure 4).

### 2.8. Development of the Machine Learning Algorithm to Assess Estrous Cycle

The 316 images of the stained smears collected during the experiment were used to create a larger dataset for better performance using the augmentation technique. Each image underwent a few changes, like random rotation between −15 and 15 degrees, horizontal flips with a 50% chance to remove cell orientation bias, and random resizing to 80% of the original size (approximately 630 × 743 pixels) to maintain important details while reducing data size. This process resulted in a dataset of 1580 images, further classified into the four stages of the estrous cycle (Diestrus 600, Estrus 400, Metestrus 380, and Proestrus 200). Finally, to speed up training and use less memory, the images were resized to a standard size of 256 × 256 pixels, ensuring that all crucial details were preserved. To identify the stage of the estrous cycle represented in an image, the dataset was divided into three components: 70% for training, 15% for validation (to evaluate the model’s performance during training), and 15% for testing. The transfer learning was employed by freezing layers to expedite the network’s training process. During training, the initial layer weights were kept fixed at zero learning rates, meaning that the frozen layer parameters were not updated. To prepare the images for training, appropriate sizes were set, and random vertical inversions and horizontal and vertical translations of no more than 30 pixels were applied to ensure that the network would not be specialized exclusively on the input data. The validation and test sets were used on initial sizes. The network parameters were improved using the “Adam Optimizer” [36]. Performance was checked every 5 iterations using validation data. The network graph was tracked during training using the “cross-entropy” evaluation metric to compare the actual distribution with the distribution predicted by the model. The model’s performance was evaluated by accuracy, precision, sensitivity, and F1 score. The corresponding equations are as follows:

(1) $$Accuracy=\frac{TN\;+\;TP}{TN\;+\;TP\;+F\;P\;+F\;N}$$

(2) $$Precision=\frac{TP}{TP+FP}$$

(3) $$Sensitivity=\frac{TP}{TP+FP}$$

(4) $$F1\;score=2\frac{Precision\;*\;Sensitivity}{Precision+Sensitivity}$$

where the abbreviations *TN*, *TP*, *FN*, and *FP* stand for True Negative, True Positive, False Negative, and False Positive classification, respectively. In the final stage of the research, the model was incorporated into a classification tool for easy use by clinicians.

### 2.9. Biochemical Analysis

The serum levels of alanine aminotransferase (ALT), aspartate aminotransferase (AST), cholesterol, triglycerides, glucose, and urea were determined spectrophotometrically using the automatic analyzer Applied Biosystem (Costa Brava, Barcelona, Spain).

### 2.10. Leptin and Insulin Assessment

The enzyme-linked immunosorbent assay (ELISA) was used to evaluate the metabolic biomarkers leptin and insulin. The ELISA technique was performed using a Biotek Microplate 50 TS washer and an 800 TS reader (Agilent Technologies Inc., Santa Clara, CA, USA). The detection protocol followed the manufacturer’s instructions from the commercial leptin (detection range 0.16–10 ng/mL, sensitivity 0.1 ng/mL, E-EL-R0582) and insulin (detection range 6.25–400 pg/mL, sensitivity 3.75 pg/mL, E-EL-R3034) kits (ElabScience, Houston, TX, USA).

### 2.11. Statistical Analysis

To determine whether the results had statistical significance, IBM SPSS Statistics software version 29.0.0.0 (IBM Corp., Armonk, NY, USA) was used. The normality of the data distribution was assessed using the Shapiro–Wilk test. For data not normally distributed, values were log-transformed and retested. If normality was achieved, parametric tests were applied; otherwise, non-parametric approaches were used. The not normally distributed data was presented as medians with interquartile ranges (25th to 75th percentiles). A two-way repeated measures ANOVA was employed to analyze body weight over time. In this model, time (day 0 to day 30) was used as the within-subjects factor, while sex (male/female) and light exposure group (CTRL, MP, PC, LED) were treated as between-subjects factors. Interaction effects (e.g., time × sex, time × group) were also evaluated. Insulin and leptin levels, measured at three time points (days 0, 15, and 30), were analyzed using the Friedman test due to the non-normal distribution of the data. Post hoc pairwise comparisons between time points were conducted using the Wilcoxon signed-rank test with Bonferroni correction. For biochemical parameters measured at the end of the experiment (e.g., ALT, AST, glucose, urea, cholesterol, triglycerides), the Kruskal–Wallis test was used to compare groups. A *p*-value < 0.05 was considered statistically significant for all analyses.

## 3. Results

### 3.1. Blue Light Exposure Influence on Body Weight

**Figure 5:**
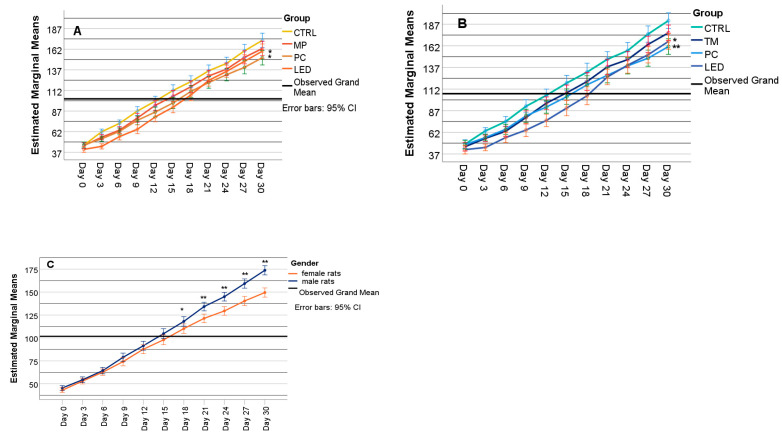
Estimated marginal means of body weight according to the group for females (**A**) and males (**B**), and according to sex (**C**) over time obtained with two-way ANOVA, where CTRL—control group with no exposure, MP—group exposed to mobile phones, PC—group exposed to the computer screen, LED—group exposed to blue light LED; * had *p*-value < 0.05 while ** had *p*-value < 0.01.

The two-way ANOVA for repeated measures included time as the within-subject factor, group (light exposure: CTRL, MP, PC, LED) (Figure 5A,B), and sex (Figure 5C) as between-subjects factors.

The test of within-subjects effects indicated that time (*p* = 0.001), sex (*p* = 0.001), time and group (*p* = 0.001), and time and sex (*p* = 0.001) significantly influenced the body weight of rats exposed to the different light sources in both females (Figure 5A) and males (Figure 5B).

The Bonferroni test for groups showed that female rats exposed to PC light (*p* = 0.010) and LED light (*p* = 0.001) had significantly lower body weights when compared to rats included in the CTRL group. Male rats exposed to PC light (*p* = 0.023) and LED light (*p* = 0.001) had also significantly lower body weights when compared to rats included in the CTRL group. An independent *t*-test was used to determine how time affected body weight differences between sexes. The results showed that females had significantly lower body weight than males from day 21 to day 30 (*p* = 0.001).

### 3.2. Estrous Cycle Identification Algorithm

Using the methodology and performance measures described in the previous section, the two networks, “Squeezenet” and “ResNet50”, were analyzed. The results are presented in Table 1.

**Figure 6:**
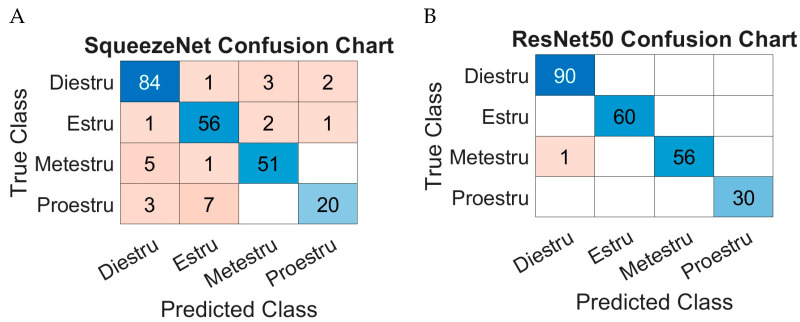
SqueezeNet confusion chart (**A**) and ResNet 50 confusion chart (**B**).

The duration of the estrous cycles observed in the rats was 4 and 5 days, respectively. During the evaluation period, two complete cycles were expected and successfully assessed per rat. For better visualization of the performances, confusion matrices are included for both models, Figure 6. By using these characteristics, a clearer overview of how well each model classifies the data can be obtained. This will show both accurate and inaccurate classifications for each class.

The results show that the “ResNet50” network outperforms the “SqueezeNet” model with an accuracy of 99% and 89%, respectively. However, both models demonstrate good performance in this field.

### 3.3. The Effect of Blue Light on the Cyclicity of the Estrous Cycle and Preputial Separation

**Figure 7:**
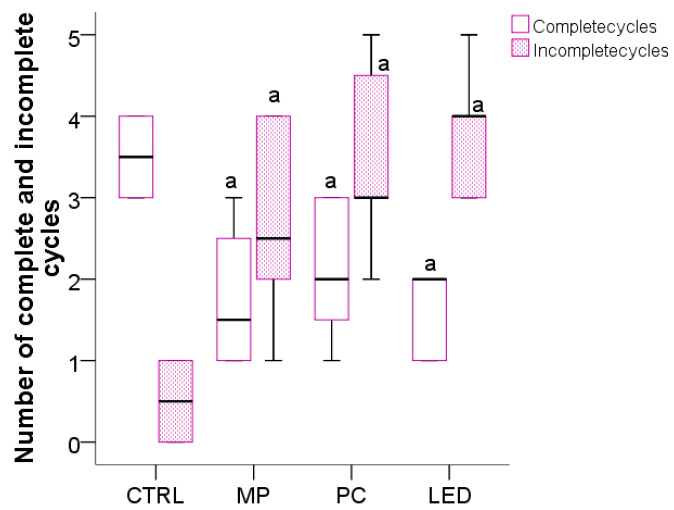
Identified estral cycling type in female rats exposed to blue light from different sources, where CTRL—control group with no exposure, MP—group exposed to mobile phones, PC—group exposed to computer screen, and LED—group exposed to blue light LED. The boxplots represent the median values (midline line), and the first and third quartiles (the extreme lines), where a had *p* < 0.05, versus the CTRL group.

An estrous cycle in rats lasts approximately 4–5 days and was identified based on the sequential presence of at least three consecutive stages, including a return to proestrus, as determined by daily vaginal smear analysis. Using this criterion, the CTRL group experienced normal conditions and displayed a high number of complete estrous cycles (over 3.5) with very few incomplete cycles (under 1) (Figure 7). Compared to the CTRL group, female rats in the MP group had significantly fewer complete cycles (*p* = 0.001) (around 2) and significantly more incomplete cycles (*p* = 0.008) (around 3). Females in the PC group had a significantly lower number (*p* = 0.01) of complete estrous cycles (over 2) and a significantly higher number of incomplete cycles (*p* = 0.001) (around 3.5) as compared to the CTRL group. The LED group also experienced a significant decrease in the number of complete estrous cycles (falling below 2) (*p* = 0.001) and a significant increase in incomplete cycles (nearly 4) (*p* = 0.001).

The age of the female rats at which the first complete estrous cycle was first noted was 33 postnatal days (PNDs) in the control group. In contrast, animals exposed to the MP and PC exhibited vaginal opening at 29 PNDs, while animals exposed to LED at 28 PNDs, indicating an accelerated onset of puberty in all treated groups compared to controls.

In males, early pseudopuberty was characterized by the separation of the foreskin. This separation occurred between 38 and 43 postnatal days (PNDs), significantly earlier as compared to the CTRL group, in which preputial separation occurred at 48 PNDs.

### 3.4. Light Effects on Metabolic Changes

**Figure 8:**
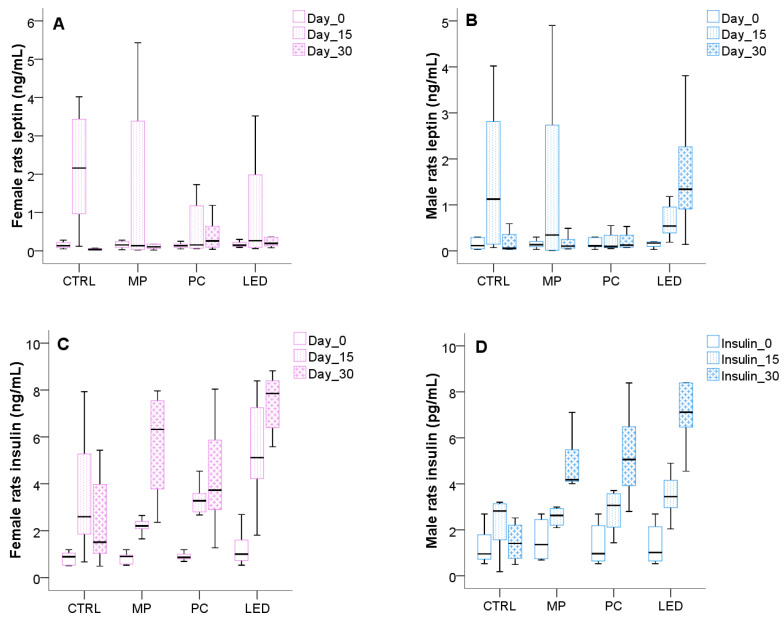
Serum concentration of leptin in the case of females (**A**) and male rats (**B**), and of insulin in the case of females (**C**) and male rats (**D**) at different time-point intervals (0–15–30 days) of exposure. Animals of both sexes were grouped according to the light exposure sources in the following groups: CTRL (had no exposure), MP (exposed to mobile phone), PC (exposed to computer screen), and LED (exposed to LED). The boxplots represent the median values (midline line) and the first and third quartiles (the extreme lines).

The serum levels of leptin and insulin are presented in Figure 8. Three time points—baseline (Day 0), midpoint (Day 15), and endpoint (Day 30)—were selected to assess both immediate and progressive hormonal responses to blue light, reflecting patterns of chronic real-world exposure. This approach also aimed to capture age-related hormonal dynamics associated with pubertal development. The Friedman test revealed a statistically significant increase in insulin levels over time across the study population (*p* = 0.001). Post hoc analysis using the Wilcoxon signed-rank test showed that insulin levels were significantly higher at Day 15 and Day 30 compared to Day 0 (*p* = 0.001), and Day 30 levels were also significantly higher than Day 15 (*p* = 0.001).

In contrast, leptin levels did not show statistically significant differences over time or between groups, according to the Friedman test, although a visible increase at Day 15 was noted in both sexes, including in the control group. This trend may reflect normal prepubertal hormonal activity, but due to sample variability, it did not reach statistical significance.

Given that insulin was the only parameter to show a statistically significant change over time, we further explored group differences using the Kruskal–Wallis test at each time point. At baseline (Day 0), no significant differences in insulin levels were observed between groups. By Day 15, female rats in the LED group had significantly higher insulin levels compared to the CTRL (*p* = 0.031) and MP (*p* = 0.001) groups. After 30 days, both female and male rats exposed to blue light showed significantly elevated insulin levels. Females in the LED group differed from CTRL (*p* = 0.001) and PC (*p* = 0.018), and females in the MP group also had higher insulin than CTRL (*p* = 0.046). Among males, all blue-light-exposed groups showed significantly increased insulin compared to controls: MP (*p* = 0.028), PC (*p* = 0.007), and LED (*p* = 0.001).

**Figure 9:**
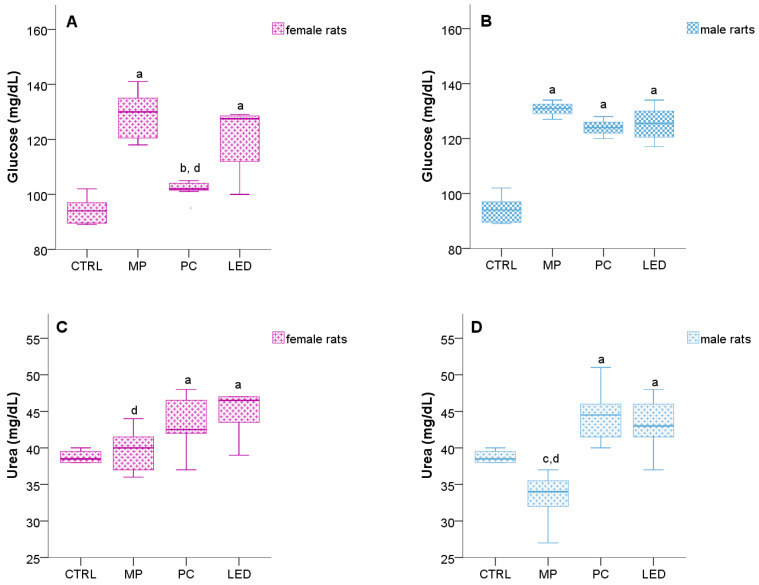

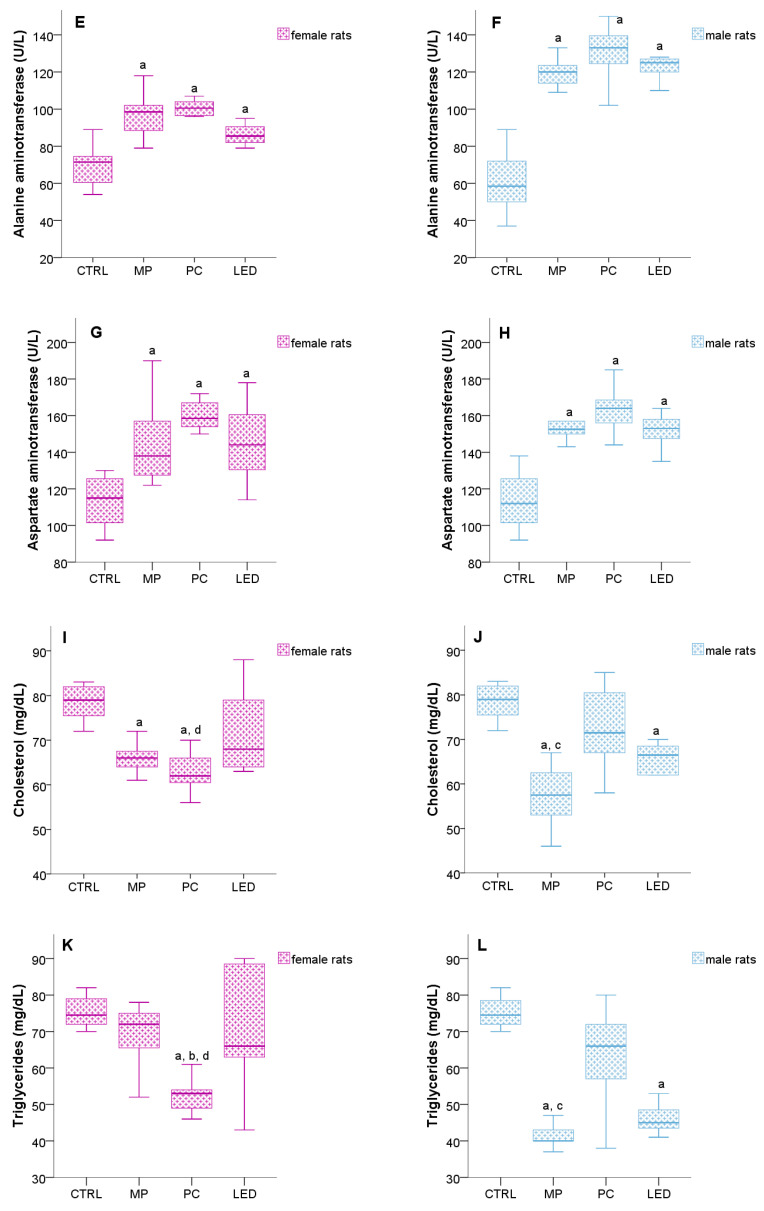
Serum concentration of glucose for female (**A**) vs male rats (**B**), urea for female (**C**) vs male rats (**D**), alanine aminotransferase for female (**E**) vs male rats (**F**), aspartate aminotransferase for female (**G**) vs male rats (**H**), cholesterol for female (**I**) vs male rats (**J**), and triglycerides for female (**K**) vs male rats (**L**) after 30 days of exposure. Animals of both sexes were grouped according to the light exposure sources in the following groups: CTRL (had no exposure), MP (exposed to mobile phone), PC (exposed to computer screen), and LED (exposed to LED). The boxplots represent the median values (midline line), the first and third quartiles (the extreme lines), a had *p* < 0.05, versus the CTRL group; b had *p* < 0.05, versus the MP group, c had *p* < 0.05, versus the PC group; d had *p* < 0.05, versus the LED group.

Thirty days post-exposure to blue light, females in the MP (*p* = 0.001) and LED (*p* = 0.001) groups displayed a significantly elevated serum glucose level compared to the CTRL group (Figure 9A). Similarly, males in all treatment groups (MP (*p* = 0.001), PC (*p* = 0.006), and LED (*p* = 0.002)) showed significantly higher serum glucose levels compared to the CTRL group (Figure 9B).

Blue light exposure resulted in significant changes in serum urea concentration. Females in the LED (*p* = 0.001) and PC (*p* = 0.013) groups exhibited a markedly elevated level compared to the CTRL group (Figure 9C). Similarly, males in the PC (*p* = 0.017) and LED (*p* = 0.042) groups displayed significantly higher concentrations as compared to the CTRL group. Rats included in the MP group had significantly lower serum urea concentration compared to the PC (*p* = 0.001) and LED (*p* = 0.001) groups (Figure 9D).

Serum alanine aminotransferase (ALT) levels were also significantly impacted by blue light exposure. Thirty days post-exposure, females in the MP (*p* = 0.001), PC (*p* = 0.001), and LED (*p* = 0.048) groups exhibited significantly higher ALT levels compared to the CTRL group (Figure 9E). Similarly, males in the MP (*p* = 0.010), PC (*p* = 0.001), and LED (*p* = 0.004) groups also showed significantly elevated ALT levels relative to the CTRL group after 30 days (Figure 9F). Serum aspartate aminotransferase (AST) concentrations were elevated in both sexes. Specifically, females in the MP (0.020), PC (*p* = 0.001), and LED (*p* = 0.011) groups displayed significantly higher AST levels compared to the CTRL group at 30 days post-exposure (Figure 9G). Similarly, males in the MP (*p* = 0.003), PC (*p* = 0.001), and LED (*p* = 0.005) groups exhibited significantly increased serum AST levels relative to the CTRL group after 30 days (Figure 9H).

Serum cholesterol levels in females from the MP (*p* = 0.005) and PC (*p* = 0.001) groups were significantly lower compared to the CTRL group after 30 days of exposure (Figure 9I). Similarly, males in the MP (*p* = 0.001) and LED (*p* = 0.006) groups exhibited significantly decreased serum cholesterol levels relative to the CTRL group. Interestingly, males in the MP group also showed significantly lower cholesterol levels compared to the PC group (*p* = 0.002) (Figure 9J).

Triglyceride levels were impacted in both sexes. Notably, females in the PC groups exhibited significantly lower serum triglyceride levels compared to the CTRL (*p* = 0.001), MP (*p* = 0.009), and LED (*p* = 0.012) groups at 30 days post-exposure (Figure 9K). Among males, the MP (*p* = 0.001) and LED (*p* = 0.001) groups showed a significantly decreased serum value relative to the CTRL group. Interestingly, males in the MP group (*p* = 0.004) compared to the PC group displayed significantly lower triglyceride levels (Figure 9L).

## 4. Discussions

This study shows that blue light exposure from different sources (LED, PC, and MP) had an impact on the onset of puberty in male and female rats. The obtained results demonstrate that, to varying degrees, exposure to light from all tested sources influenced the timing of puberty onset and induced significant metabolic alterations. These included a reduction in growth rate, decreased insulin sensitivity, lowered cholesterol and triglyceride levels, and increased serum levels of liver enzymes ALT and AST. Such changes suggest a systemic response to chronic light exposure, which may interfere with normal endocrine and metabolic regulation during critical developmental periods. These findings align with the primary objective of the study, which was to assess the potential impact of prolonged blue light exposure on puberty onset and associated metabolic parameters. Further, these results are consistent with previous studies that reported similar effects. For example, Uğurlu et al. (2023) showed that male rats exposed to 6 or 12 h per day of blue LED light (450–470 nm) exhibited significantly earlier puberty compared to controls, along with histopathological changes in the testes [31]. In a parallel study, the same group demonstrated that female rats under similar blue light exposure also experienced precocious puberty, accompanied by hormonal alterations such as increased LH and estradiol levels [29].

In the case of females, early vaginal opening and early preputial separation for male rats [33] indicated the early onset of PP, similarly to this study. As seen by the cyclicity of the estrous cycles, female rats exposed to MP, PC, and LED had a significantly altered number of both complete and incomplete estrous cycles. Thus, exposure to blue light could also be linked to the time of the first estrous cycle and the length of the estrous cycle. The association between external factors like endocrine-disrupting chemicals has been previously associated with the time to first estrous cycle and its length [37].

Both human and experimental models exhibit a natural circadian rhythm characterized by a 12:12 h light/dark cycle. Disruptions to this rhythm, such as extended exposure to artificial light, variations in light intensity, or alterations in light color, can induce significant stress and lead to various metabolic disturbances [38]. The suprachiasmatic nucleus (SCN), the principal circadian regulator, coordinates daily rhythms in food intake through interactions with the arcuate nucleus of the hypothalamus [39]. The SCN also projects to the lateral habenula, a brain region implicated in regulating mood, reward, and feeding behaviors. The lateral habenula, in turn, receives input from the lateral hypothalamic area, which plays a key role in feeding and reward processing. Additionally, the paraventricular nucleus of the hypothalamus (PVN) of the hypothalamus projects to the spinal cord’s intermediolateral column, thereby regulating pineal melatonin secretion [40]. Sympathetic and parasympathetic projections also extend to peripheral organs such as the pancreas [41] and liver [42], further linking the central clock to metabolic regulation. Conversely, extensive research documented in the literature demonstrates that disruption of the circadian system, particularly the desynchronization between central and peripheral clocks, increases metabolic risk [43,44,45]. Extensive research has shown that circadian system disruption—particularly desynchronization between central and peripheral clocks—significantly increases metabolic risk [46,47,48,49]. Therefore, examining alterations in lipid profiles offers a valuable approach to understanding blue light-induced circadian disturbances and their metabolic consequences. In this context, alterations in lipid profiles play a significant role in further investigating blue light-induced circadian disturbances.

This study further supports this notion by revealing significant decreases in both cholesterol and triglyceride levels in both sexes compared to controls. It is already known that lipid concentrations vary greatly between pubertal stages and experience large, sex-specific variations during physical growth and sexual maturity [46]. A significant increase in the need for cholesterol, which leads to a decline in lipid levels in response to the metabolic demands of early puberty, may be the cause of this change [46,47]. In this study, cholesterol levels decreased in females exposed to MP and PC light, while males showed reductions in MP and LED groups, indicating different lipid metabolism responses. Triglyceride reductions were prominent in males exposed to MP and LED, whereas females exhibited this change only in the PC group. These variations, which were different among male and female rats, could be explained by both hormonal changes and metabolic adjustments induced by early puberty, which are gender specific.

Further, Leptin is a key hormone that regulates satiety signals involved in energy homeostasis and the regulation of appetite, also known for its role in puberty initiation [48]. Several studies have demonstrated leptin’s promotion of fatty acid oxidation and lipolysis stimulation [49,50]. While previous studies have implicated leptin as a potential mediator in lipid metabolism during pubertal transitions [51], our findings did not reveal statistically significant changes in leptin levels across experimental groups, despite noticeable fluctuations in some subgroups. A transient increase in leptin concentration at Day 15 was observed in both male and female control animals, and is consistent with previously documented physiological rises in leptin during the prepubertal phase [52]. Thus, leptin acts as a permissive signal for hypothalamic activation and pubertal onset, and its transient elevation may reflect normal developmental processes. However, since no statistically significant leptin changes were detected, the observed reductions in cholesterol and triglyceride levels in blue light-exposed animals cannot be conclusively linked to leptin in this context. Thus, in these experimental conditions, leptin is unlikely to mediate blue-light-induced pubertal changes. Further research with larger sample sizes or longer exposure durations may clarify leptin’s role in blue-light-induced metabolic shifts [49].

Next, extended blue light exposure could induce stress and anxiety in rats, evidenced by altered body weight. This hypothesis is supported by several other studies, which suggested that weight decrease in rats could appear due to anxiety-induced behaviour, possibly due to increased physical activity, basal metabolism, and adaptive thermogenesis [53,54].

Further, insulin is the neuroendocrine hormone regulated by the hypothalamus and pancreas that maintains normal glucose homeostasis at the level of all body tissues and represents an important factor in the pseudopuberty process [32,55]. Our study found that prolonged exposure to blue light from digital devices significantly affected insulin levels in both male and female rats. These effects were not immediate but developed progressively during the 30 days. By Day 15, insulin levels were significantly elevated in female rats exposed to LED light compared to controls, and by Day 30, both sexes exhibited marked insulin increases in response to blue light, particularly in the MP, PC, and LED groups. These results suggest that chronic blue light exposure may disrupt glucose metabolism through endocrine dysregulation, as evidenced by elevated insulin levels and altered cholesterol profiles. Specifically, cholesterol levels decreased in females exposed to MP and PC light, while males showed reductions in MP and LED groups, indicating that this could contribute to the early onset of puberty and associated metabolic imbalances [56,57]. During premature puberty, insulin’s action can cause abnormalities in serum glucose, cholesterol, triglycerides, and body weight. Insulin is essential for controlling glucose and lipid metabolism. Also, disruptions in insulin secretion in rodents initiate diabetes, suggesting a strong link between the circadian timing system and pancreatic beta cell function [58]. Within our study, the disruption of CR can impair insulin sensitivity, thus contributing to elevated glucose and insulin levels in these rats.

Alteration of the CR by light pollution induced alterations in hepatic and renal function, as evidenced by elevated levels of AST, ALT, and sex-specific changes in serum urea. These findings suggest that CR disruption may affect the delicate balance of metabolic processes, leading to organ dysfunction.

More exactly, within this study, both male and female rats exhibited disruptions in glucose, cholesterol, triglyceride, and urea levels, the patterns varying significantly by sex and light source. For instance, glucose levels increased across all light-exposed groups in males, but only in the MP and LED groups in females, suggesting broader sensitivity to blue light in males regarding glycemic regulation. Additionally, serum urea levels increased in both sexes after PC and LED exposure, yet only male rats in the MP group had a significant decrease, highlighting an inverse sex-specific response. These sex-specific effects may be attributed to differences in hormonal regulation and circadian rhythm dynamics between males and females [59,60]. Previous studies have shown that endocrine responses to environmental stressors, including light exposure, are modulated by sex hormones, which influence both metabolic and neuroendocrine pathways [61,62,63]. Also, these differences suggest that the physiological impact of blue light is modulated by both biological sex and the spectral or intensity characteristics of the emitting device, and emphasize the importance of addressing the public health guidance concerning screen exposure based on age and sex.

Overall, the impact of blue light exposure on metabolic responses in rats raised multiple hypotheses that further need confirmation. One would refer to the fact that the complex hormonal regulation during pubertal development is reflected in lower body weight and impaired glucose metabolism. Further, the overall metabolic disturbance observed in the rats could potentially be linked to insulin resistance. Finally, the metabolic alterations observed in rats with induced precocious puberty—reduced cholesterol and triglycerides, decreased body weight, and increased glucose and insulin could be explained by disruptions in CR induced by prolonged blue light exposure.

## 5. Study Limitation

Our study has several limitations. First, the selected duration of blue light exposure may not have been sufficient to comprehensively evaluate the restoration of estrous cyclicity and the attainment of full sexual maturity in all female subjects. A prolonged exposure period might be required for a more thorough assessment. Second, the daily vaginal cytology sampling schedule may have overlooked transient stages of the estrous cycle due to their brief duration. Although we sought to mitigate this limitation by collecting samples consistently at the same time each day, a more frequent sampling protocol may yield a more complete characterization. Furthermore, the inability to quantify food intake, as animals had ad libitum access to food, restricts our capacity to establish a definitive causal link between blue light exposure and changes in body weight. Also, the absence of testosterone measurement in male rats limits our ability to comprehensively assess endocrine changes associated with blue light-induced precocious puberty. Additionally, a notable limitation of this study is the inability to measure the light intensity of the other two light sources to which the animals were exposed throughout the experiment. Future studies incorporating food intake monitoring and precise light intensity measurements may provide more conclusive insights.

## 6. Conclusions

Our findings indicate that 30 days of exposure to blue light emitted from various devices may contribute to the early initiation of puberty and the length of the estrous cycle, inducing irregular cyclicity among female rats. Also, prolonged blue light exposure acts as a stressor, inducing significantly lower body weight gain, and lower cholesterol and triglyceride values. Also, glucose and insulin regulation were modified after exposure to blue light, resulting in elevated serum levels in both females and males. Overall, the data suggests potential metabolic disruptions, evidenced by altered serum lipid and carbohydrate profiles, alongside variations in body mass.

## Figures and Tables

**Table 1:** Model performance metrics for SqueezeNet and ResNet50.

	Metrics	Diestrus	Estrus	Metestrus	Proestrus	Mean
SqueezeNet	Accuracy	0.9333	0.9333	0.8947	0.6657	0.8903
	Precision	0.9032	0.8615	0.9107	0.8696	0.8863
	Sensitivity	0.9333	0.9333	0.8947	0.6667	0.9956
	F1 score	0.9180	0.8960	0.9027	0.7547	0.8679
ResNet50	Accuracy	1	1	0.9825	1	0.9956
	Precision	0.989	1	1	1	0.9973
	Sensitivity	1	1	0.9825	1	0.9956
	F1 score	0.9945	1	0.9912	1	0.9964

## Data Availability

The datasets generated and/or analysed during the current study are available in the [Mendely Data] repository, [https://data.mendeley.com/datasets/yrxp6gpjw2/1, accessed on 9 April 2025].
